# Assessing springtime vertebrate prey of sympatric mesopredators in the southeastern United States using metabarcoding analysis

**DOI:** 10.1371/journal.pone.0293270

**Published:** 2023-10-25

**Authors:** Jordan L. Youngmann, Stacey L. Lance, John C. Kilgo, Charles Ruth, Jay Cantrell, Gino J. D’Angelo

**Affiliations:** 1 Daniel B. Warnell School of Forestry and Natural Resources, University of Georgia, Athens, Georgia, United States of America; 2 Savannah River Ecology Laboratory, University of Georgia, Aiken, South Carolina, United States of America; 3 United States Department of Agriculture Forest Service, Southern Research Station, New Ellenton, South Carolina, United States of America; 4 South Carolina Department of Natural Resources, Columbia, South Carolina, United States of America; Zoological Survey of India, INDIA

## Abstract

Coyotes (*Canis latrans*) colonized the eastern United States over the last century and formed a 3-species predator guild with bobcats (*Lynx rufus*) and gray foxes (*Urocyon cinereoargenteus*) across much of the southeastern United States. Diets among the three species vary along with respective impacts on game species such as white-tailed deer (*Odocoileus virginianus*) and wild turkeys (*Meleagris gallopavo*). To determine predation impacts on vertebrate prey and dietary overlap in consumption of prey items, we assessed diets of coyote, bobcat, and gray fox during spring, coinciding with white-tailed deer fawning and wild turkey nesting and brood rearing. We sampled across three sites along the Savannah River in South Carolina from mid-May through mid-June of 2020–2021. We collected 180 scat samples along 295.9 kilometers (71.1–122.4 km/site) of unpaved secondary roads and used DNA metabarcoding to determine vertebrate diet items. We identified predator species of scat using DNA metabarcoding and species-specific mtDNA fragment analysis (153 were coyote, 20 bobcat, and seven gray fox). Overall, we found evidence that two species, coyote and bobcat, consumed deer while all three consumed turkeys. Frequency of deer in the diet varied across sites for coyotes from 62–86% and wild turkey was present with a frequency of occurrence of 9% for coyotes, 5% for bobcats, and 14% for gray fox. Vertebrate diet specialization was evident across predator species with high frequency of deer in coyote diets, rabbits and small mammals in bobcat diets, and herpetofauna in gray fox diets. During deer fawning and wild turkey nesting and brood rearing, dietary overlap appears to be mediated by disparate selection of prey items, which reduced competition among coyotes, bobcats, and gray foxes. Use of DNA metabarcoding may augment our understanding of dietary preferences within this predator guild by providing increased resolution of diet composition among important game species.

## Introduction

Coyotes (*Canis latrans*), bobcats (*Lynx rufus*), and gray foxes (*Urocyon cinereoargenteus*) are mesopredators comprising a three-predator guild that has been well-documented across their sympatric range [1–4]. Range overlap among these species has increased over the last century as coyotes have colonized the eastern United States [5, 6], in part due to the extirpation of top predators such as the red wolf (*Canis rufus*) and eastern cougar (*Puma concolor*). In areas lacking larger predators such as black bears (*Ursus americanus*), coyotes and bobcats are apex predators within the trophic hierarchy of the eastern United States, due to their size and consumption of ungulates such as white-tailed deer (*Odocoileus virginianus*) [3, 7, 8]. The success of coyotes in the eastern United States has implications for other species, including other mesopredators. For example, recent research has documented marked declines in gray fox populations in the region, potentially as a result of exploitative and interference competition with colonizing coyote populations including overlap in diet and spatial exclusion [4, 9, 10].

Assessing diets is an important step towards understanding how coyotes, bobcats, and gray foxes compete, and how this predator guild influences the food web in the eastern United States. While many studies have reported considerable overlap in space use and diet among coyotes, bobcats, and gray foxes [2, 4, 11–16], differences in hunting strategies and differing trophic functions may potentially alleviate intraguild competition. For example, bobcats are obligate carnivores that hunt via ambush tactics, whereas coyotes and gray foxes are generalist omnivores that hunt cursorily and supplement their diet with non-animal foods [1, 17–22]. Bobcat diets in the eastern United States are largely comprised of small mammals, including squirrels (*Sciurus* spp., *Glaucomys* spp., *Sigmodon hispidus*), lagomorphs, and white-tailed deer [13, 22, 23]. Coyotes in the eastern United States tend to exhibit greater diversity in food choice than bobcats, consuming white-tailed deer, small mammals such as the cotton rat (*Sigmodon* spp.), lagomorphs, soft mast including *Rubus* spp. and persimmons (*Diospyros virginiana*), and insects [13, 17, 19–21, 24, 25]. There has been little research conducted on gray fox diets in the eastern United States, especially since coyote colonization, but studies in the western United States have shown them to be generalist omnivores with a diet that is similar to coyotes [26–28]. Recent stable isotope analysis has shown overlap in the diet of coyotes and gray foxes in the eastern United States [4, 29]. Through assessment of the vertebrate species found in coyote, bobcat and gray fox diets, we hope to better understand how this predator guild interacts and its role in shaping predator-prey dynamics in the eastern United States.

In the southeastern United States, the coyote-bobcat-gray fox predator guild is thought to be negatively impacting two important game species: white-tailed deer and wild turkey (*Meleagris gallopavo*). Thus, assessing the diet of all three predator species will provide data on intra-guild competition as well as inform management of game species. Population trajectories of white-tailed deer have stabilized or declined in some southeastern states, coincident with rises in coyote populations [30]. Past studies have reported high rates of white-tailed deer fawn mortality due to predation, especially in the southeastern United States [31–34]. Additionally, coyote, bobcat, and gray fox depredation of turkey hens and poults during nesting and brood rearing periods [35–37] C. Ruth, South Carolina Department of Natural Resources, personal communication] may be partially responsible for declining wild turkey populations over the last decade [38–41]. Although these predators may predate hens, particularly during the spring [35–37, 42–44], there is scant evidence across diet studies that they rely on wild turkeys as a food source [12, 15, 45]. Better understanding of predator impacts on populations of wild turkeys and white-tailed deer is vital for agencies to effectively conserve and manage predator and prey species alike.

Diet analyses have traditionally used morphometric identification of remnant prey items in either scat or stomach contents [46]. However, analysis of both host and prey species through visual identification of scat is often inaccurate [13, 47]. Morin et al. [13] documented high rates of misclassification of coyote and bobcat scat, which led to incorrectly attributing diet to the wrong predator. Although some recent studies have used genetic methodologies [11, 14, 29, 48, 49], Monterroso et al. [47] identified only 8% of 400 diet studies that used genetics to identify predator species. Morphometric identification of diet items within scat also can be biased because of differences in digestion efficiency and the inability to classify trace amounts of remnant prey items. For instance, traditional diet analyses have typically failed to show substantial predation on avian species by coyotes, bobcats, or gray foxes, even during periods when ground nesting birds are on their nests [23, 50]. However, coyotes, bobcats, and gray foxes do appear to have consistent, but low, presence of avian species in their diets [26, 27, 51, 52]. The general lack of avian species in scat studies may be due to low predation rates or the inability of morphometric diet analyses to identify avian remains. Several methodological studies have shown disparities in identification of avian remains between analysis of stomach contents and morphometric scat analysis, possibly due to differential rates of digestion [53, 54]. To address past shortcomings in sampling methods, we used DNA metabarcoding, which uses genetic sequencing to identify both the host and varied prey species contained within each scat sample. This method may provide further resolution in determining the dietary composition of each species [47, 55, 56] and has recently been used successfully to study coyote diets [57, 58]. DNA metabarcoding may allow better understanding of how coyotes, bobcats, and gray foxes function as competitors within their guild and as apex predators on the landscape.

To assess intraguild competition among coyotes, bobcats, and gray foxes and to better understand predation levels on game species, we sampled scat during the fawning period of white-tailed deer and nesting of wild turkeys. Our objective was to compare vertebrate diet items using metabarcoding analysis among these sympatric predators to assess the potential for competition among intraguild interactions in the southeastern United States. We hypothesized that coyotes and gray foxes would exhibit higher diversity in their diets than bobcats due to their generalist diet preferences. Additionally, we hypothesized that we would observe higher levels of dietary overlap between coyotes and gray foxes due to their similar diets and hunting strategies. We also predicted that because genetic methods can be more precise than visual identification of prey items [13, 47, 55, 56], we would find higher frequency of avian prey within predator diets than previously reported.

Furthermore, because coyote scat analysis can be used to assess the influence of landcover covariates on coyote diet [20, 21], we sought to replicate Hinton et al. [21] to address three additional hypotheses. Firstly, forest cover would positively affect the consumption of deer and wild turkeys. Forest cover is a predominant landcover type within our study region and previous research has documented increased predation of fawns in relation to forest patch size and availability, although causality has been difficult to quantify [59, 60]. Limited understory vegetation in closed canopy forests should increase the ability of coyotes to find fawns and turkeys. Secondly, Julian date would positively influence consumption of fawns as previously reported in the southeastern United States [32, 61, 62]. Finally, consumption of other prey items would be negatively correlated with the consumption of deer and wild turkeys. Coyotes exhibit prey switching behavior to optimize foraging by capitalizing on high quality resources and fawns experience heavy predation rates in the springtime, likely due to their availability and vulnerability [20, 21].

### Study area

**Fig 1:**
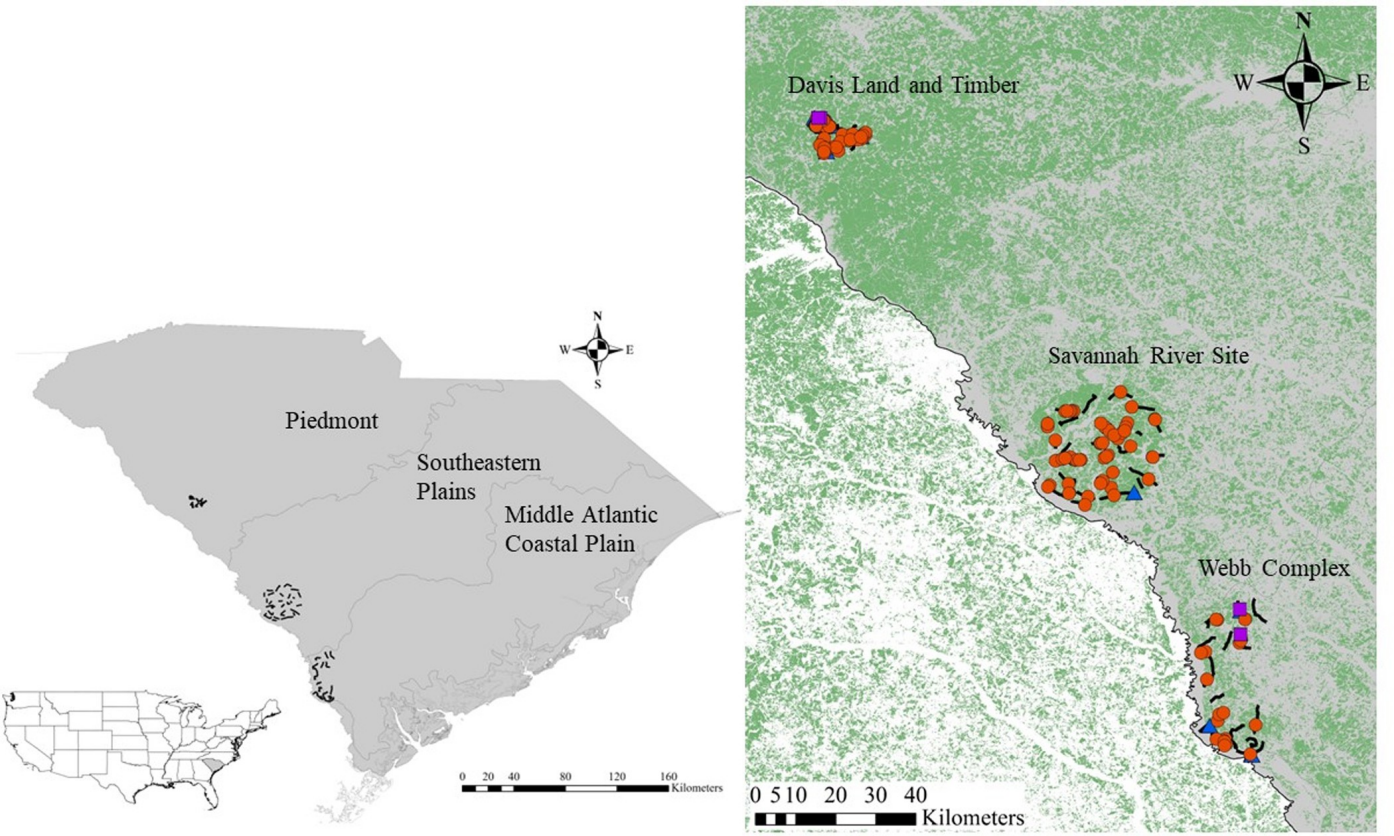
Study sites along the Savannah River, South Carolina, USA where scat was collected to assess diet of coyotes (*Canis latrans*, orange circles), bobcats (*Lynx rufus*, blue triangles), and gray foxes (*Urocyon cinereoargenteus*, purple squares) during May-June 2020–2021. Inset shows sample transects used to collect scat for diet analysis and forest cover derived from the National Land Cover Database 2019 used to model landcover impacts on coyote diet.

We assessed diets of coyote, bobcat, and gray fox populations in three Level III ecoregions in South Carolina, USA: the Davis Land and Timber property in the Piedmont, the U.S. Department of Energy’s Savannah River Site in the Southeastern Plains, and the South Carolina Department of Natural Resources’ Webb Complex in the Middle Atlantic Coastal Plain (Fig 1) [63]. The Davis Land and Timber property is entirely privately owned, Savannah River Site is a National Environmental Research Park, and the Webb Complex is a mixture of state-owned wildlife management areas and privately owned lands. The Piedmont ecoregion lies between the Blue Ridge Mountains and the Southeastern Plains. Originally dominated by oak-hickory-pine (*Quercus-Carya-Pinus*) forests, the Piedmont has experienced extensive cotton, corn, tobacco, and wheat farming [64]. However, much of the region is now covered by both natural and planted pine stands. Mean annual temperature in the Piedmont is approximately 15°C with a mean annual precipitation of 1229 mm [65]. The Southeastern Plains is typified by sandy soils and was comprised historically of mostly longleaf pine (*Pinus palustris*) forest although it now contains extensive amounts of cultivated cropland and pasture/hay with large areas of pine plantations [64]. However, the Savannah River Site is almost entirely forested in planted pine, with bottomland hardwoods scattered throughout [66]. Mean annual temperature in the Southeastern Plains is ~16°C with a mean annual precipitation of 1358 mm [65]. The Middle Atlantic Coastal Plain contains lowland plains filled with swamps, marshes, and estuaries. Also originally covered in longleaf pine, many areas have been converted to pine plantations [64]. Mean annual temperature in the Middle Atlantic Coastal Plain is ~15.5°C with a mean annual precipitation of 1229 mm [65].

## Methods

### Ethics statement

Our research only involved the non-invasive collection of scat with no live capture, handling, or killing of animals. We were, therefore, exempt from the University of Georgia Institutional Animal Care and Use Committee protocols.

### Sampling methods

We sampled each site from mid-May through mid-June during 2020 and 2021, coincident with fawning in white-tailed deer and nesting in wild turkeys within this region [32, 67]. We established 71.1 km of transects at Davis Land and Timber, 122.4 km at Savannah River Site, and 102.4 km at the Webb Complex. We conducted our sampling along secondary dirt and gravel roads that were evenly spaced and limited in vehicle traffic. We collected scat on the first day and then every three days for 10 total sampling days over a 31-day period. This ensured that each scat collected was < 3 days old except for the first day of collection. We conducted each sampling pass by driving a vehicle along each transect at ~8 km/hr looking for scat.

We used a new wooden sampling stick or sterilized forceps to place an approximately 0.4-mL cross-section of scat into a 2 mL tube containing 1.6 mL of DETs (DMSO/EDTA/Tris/salt) buffer [68]. We recorded the sample ID, GPS coordinates, and appearance (i.e., consistency, color, contents, etc.). We placed the remaining scat into a sealed plastic bag marked with the date, GPS coordinates, and sample ID. We stored buffered samples at ambient temperature. We extracted DNA from each sample using Qiagen’s QIAamp DNA Stool Mini Kit (Qiagen, Valencia, California, USA) and methodologies were conducted in two Qiagen QIAcubes. We followed Qiagen’s extraction protocols except for the final step where we eluted with 50 μl Buffer ATE and then pipetted each sample by hand back onto the filter and spun the elution product back through the filter tube to ensure that all DNA was captured from the extraction process. We then sent extracted samples to Jonah Ventures (Boulder, CO) for metabarcoding sequencing and analysis.

### Metabarcoding analysis

Although coyotes and gray foxes are recognized as generalist omnivores, we only assessed mesopredator diets using a vertebrate metabarcoding primer. Kluever et al. [57] recently reported that metabarcoding analysis of coyotes in Florida resulted in inconclusive plant diet due to the inability to ascertain how plant DNA may have ended up in scat. They noted that the predominate plant species detected was *Pinus* spp., potentially due to pollen/seeds/spores deposition. As our sampling period was during the growing season in the Southeastern United States and samples were likely subjected to significant pollen deposition and our primary research objective was to document predation by mesopredators on vertebrate prey species, we decided to only assess vertebrate prey items. However, we recognize this minimizes any inference we might make concerning dietary overlap among bobcats, coyotes, and gray foxes.

We sent extracted DNA to Jonah Ventures for library preparation, sequencing, and bioinformatic analysis. Jonah Ventures used a segment of the Actinopterygii rRNA 12S gene (Ac12S) for PCR amplification with forward and reverse primers containing a 5’ adaptor sequence for indexing and Illumina sequencing [69]. The 25 μL PCR reactions followed Promega PCR Master Mix specifications (Promega catalog # M5133, Madison, WI), including 12.5ul of Master Mix, 0.5 μl of each primer, 1.0 μl of gDNA, and 10.5 μl of DNase/RNase-free H_2_O. The PCR cycling conditions started with denaturation at 94°C for 3 minutes, followed by 45 cycles of 30 seconds at 94°C, 30 seconds at 52°C, and 1 minute at 72°C, and a final elongation at 72°C for 10 minutes. Amplicons were cleaned through incubation with Exo1/SAP for 30 minutes at 37°C followed by inactivation at 95°C for 5 minutes and stored at -20°C. To incorporate Illumina adaptors and individual sample barcodes they performed a second round of PCR using Promega Master mix, 0.5 μM of each primer and 2 μl of cleaned DNA from the first PCR reaction. The second PCR cycling conditions consisted of an initial denaturation of 95°C for 3 minutes followed by 8 cycles of 95°C for 30 seconds, 55°C for 30 seconds and 72°C for 30 seconds. To standardize sample concentrations before sequencing the indexed amplicons were cleaned and normalized using SequalPrep Normalization Plates (cat#A10510-01; Life Technologies, Carlsbad, CA) and following manufacturer’s protocols. The final pool consisted of 5μl of each normalized sample. Jonah Ventures conducted three independent replicates of PCR to increase the likelihood of identifying prey sequences.

Sample library pools were sequenced on an Illumina MiSeq (San Diego, CA) in the Colorado University Boulder BioFrontiers Sequencing Center using the v2 500-cycle kit (cat# MS-102-2003). All bioinformatic processing was done by Jonah Ventures and followed the methods detailed in Craine [70]. In general, sequences were demultiplexed, primers removed, and low-quality reads were discarded. Then taxonomy was assigned to each ESV by mapping them against GenBank reference data [71] and Jonah Ventures voucher sequences records. The consensus taxonomy was generated by first considering 100% matches, and then going down in 1% steps until matches were present for each ESV [70].

### Data preparation

To prepare data for analysis, we first removed sequences that contained base pair matches with reference databases at less than 90% and any samples that contained 0 reads. We then identified the likely host species as sequences that contained the greatest reads within a sample. In cases where a clear predator species had fewer reads than a concurrent prey species, we identified the host species as the predator. We then matched host predator species identified through the metabarcoding sequence with an independent species identification methodology using a mitochondrial DNA (mtDNA) control-region multiplex described in De Barba et al. [72]. We included this step due to the occurrence of rare cases of introgression between coyotes and domestic dogs during colonization of the Eastern United States and some admixture remains in the region [73, 74]. We used the independent, mitochondrial species identification to verify coyote, bobcat, and gray fox samples and to rectify discrepancies between samples identified as coyote and those identified as domestic dog. Specifically, in cases where metabarcoding sequences identified as domestic dog, but mtDNA identified as coyote, we assigned that sample as coyote. Similarly, in cases where metabarcoding sequences identified a sample as coyote, but mtDNA identified as domesticated dog, we assigned that sample as coyote. Cases of admixture between coyotes and domestic dogs should be most prevalent in coyote individuals, not free-roaming dogs. We assumed that evidence of admixture in our samples (i.e., confusion between metabarcoding and mtDNA species identification) is more likely found in coyote samples, not domestic dog samples. In a few cases, multiple predators were identified within a sample, with no clear indication of which was the host species based on the number of metabarcoding reads present or mismatches with mtDNA identification. Therefore, we removed those samples as cases of unknown predators. Finally, we removed or rectified spurious sequence identifications that either did not match a known endemic species or could be clearly assigned to a known endemic species.

### Statistical analysis

#### Frequency of occurrence

Frequency of occurrence (FO) is a readily used metric for analyzing diets by averaging the occurrence of an individual prey species across all samples [27, 46, 75, 76]. To calculate FO, we first categorized prey species into eight major groups: deer, wild turkey, lagomorphs, squirrels, small mammals (*Microtus* spp., *Sigmodon hispidus*, etc.), birds, herpetofauna, and other. For each predator we divided the total occurrence of each group by the number of samples collected and multiplied by 100 to present FO as a percentage. For comparison among sample sites, we conducted chi-squared contingency table analysis using absolute FO as described in Wright [77] to avoid pseudoreplication.

#### Prey diversity and dietary overlap

We used a suite of ecological indices to compare diet diversity and overlap among the predator guild in our study. Due to low sample sizes for both bobcat and gray fox, we were only able to compare diet diversity and overlap among sample sites for coyotes. We used uniquely identified prey species to calculate a paired differences index (PDI), specialized diet (*d*), and species specificity index in the package ‘bipartite’ in Program R [78, 79] to assess the breadth and specificity of predator diets. Diet specialization of a species can be estimated using PDI and ranges from 0 (generalist) to 1 (specialist) [80, 81]. Estimates of *d* assess the degree a predator relies on a single prey item as opposed to a random selection of available prey wherein 0 denotes no specialization and 1 denotes complete specialization [82]. Using the eight FO categories of prey items, we estimated Shannon’s Diversity Index and Pianka’s index of niche overlap (*O*) in the package ‘spaa’ to assess dietary partitioning and explore the possibility of exploitative competition among sympatric predators [83].

### Landcover models

Ward et al. [20] used GPS-collared individuals to identify core areas of coyote pack home-ranges. They then systematically sampled core areas for scat to relate diet composition to pack-level landcover covariates. Resident coyote packs showed limited home-range overlap and by pooling scat across pack core areas, Ward et al. [20] avoided pseudoreplication through the non-independence of scat from the same individual or social group. Hinton et al. [21] was unable to identify pack home-ranges, but instead used a home-range estimator to identify clustering across their scat locations. Hinton et al. [21] used these clusters as a heuristic for individual pack ranges and modeled the influence of canopy cover on coyote diet with scat clusters used as a random variable to account for pseudoreplication.

In order to identify areas of clustered scat locations similar to the methodology described in Hinton et al. [21], we calculated 50% kernel density estimates (KDEs) with the h-plugin smoothing parameter using the ‘adehabitatHR’ package for R (Version 3.6.3) [84]. We limited our models to coyote scat due to low sample sizes in both bobcat and gray fox samples. We censored scat outside of our 50% KDEs and modeled turkey and deer presence in scat as a binomial response variable of 1 or 0. Our explanatory variables included mean forest cover across each KDE cluster calculated from the 2019 National Land Cover Database (NLCD) [85] by grouping the deciduous forest, mixed forest, and coniferous forest landcover groups together. We also included the Julian date of scat collection as a continuous variable along with FO of prey including deer, wild turkeys, rabbits, small mammals, and birds within each KDE cluster. We removed squirrels and herpetofauna from our analysis due to the low presence found within our scat samples. Finally, we included both the individual KDE cluster and the sample site as random variables to account for pseudoreplication. We constructed three groups of generalized linear mixed models (GLMMs) to address the influence of forest cover, Julian date, and other diet items on turkey and deer consumption. We conducted our analysis in the package lme4 in Program R [86] and used Akaike’s information criterion adjusted for small sample sizes (AICc) to select the best approximating models [87].

## Results

We collected 222 scat samples during the spring of 2020 and 192 scat samples during the spring of 2021 for a total of 414 samples. After removal of samples containing no purported prey species, no reads, or reads below our 90% identity threshold, we assessed 208 samples collected across our three sample sites (50.2% of total samples collected). Using both metabarcoding reads and mitochondrial species assignment, we found consensus identification of predator for 20 bobcats, 153 coyotes, and seven gray foxes (Fig 1 and Table 1). One bobcat sample and 10 coyote samples were missing peaks for mtDNA identification and were only identified from metabarcoding sequences, and nine coyote samples were identified as dog through metabarcoding but as coyote through mtDNA. All gray fox samples were identified through both metabarcoding and mtDNA methods. In addition, we identified 10 domestic dog samples through both metabarcoding and mtDNA methods and two raccoon samples using metabarcoding sequences. We did not identify any red fox (*Vulpes vulpes*) samples across our three sites for either 2020 or 2021. Finally, we were unable to distinguish among purported predator species for 16 samples due to a combination of mixed metabarcoding reads and mitochondrial identification and therefore removed these samples from further analysis. Due to low sample sizes for bobcats and gray foxes, we pooled all sites and years across species to conduct comparisons among predators while using only coyote samples to compare among sites.

**Table 1:** Sample sizes of scat used for dietary analysis at sites along the Savannah River in South Carolina, USA for three mesopredators: Coyotes (*Canis latrans*), bobcats (*Lynx rufus*), and gray foxes (*Urocyon cinereoargenteus*) during May-June 2020–2021.

Predator	Savannah River Site	Davis Land and Timber	Webb Complex	Total
**Bobcat**				
2020	1	5	3	9
2021	0	9	2	11
**Coyote**				
2020	21	43	5	69
2021	37	31	16	84
**Gray Fox**				
2020	0	5	2	7
2021	0	0	0	0

### Diets across predator species

#### Frequency of occurrence

**Fig 2:**
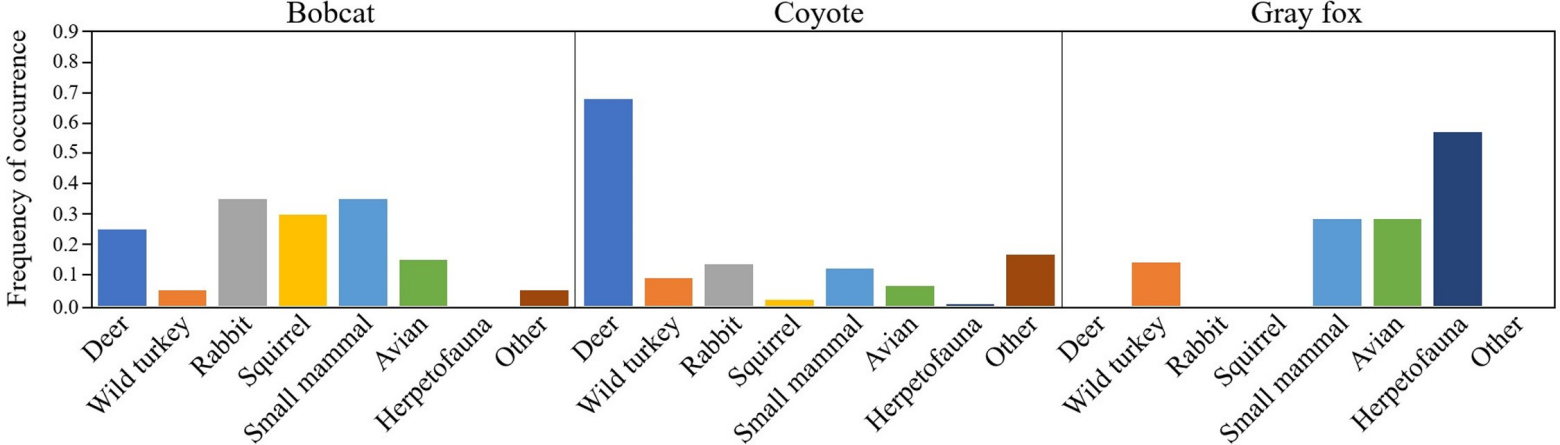
Frequency of occurrence for prey items of coyotes (*Canis latrans*), bobcats (*Lynx rufus*), and gray foxes (*Urocyon cinereoargenteus*) along the Savannah River, SC during May-June 2020–2021.

We found that coyote samples exhibited the highest FO for deer consumption across sample sites and years (68.0%), followed by bobcats (25.0%), while gray foxes showed no consumption of deer (Table 2 and Fig 2). All predators consumed wild turkeys, with gray foxes having the highest FO at 14.3%, followed by coyotes (9.2%) and bobcats (5.0%). Bobcats had the highest FO of lagomorphs (35.0%), followed by coyotes (13.7%), while gray foxes showed no consumption of lagomorphs. Similarly, only bobcats (FO = 30.0%) and coyotes (FO = 2.0%) consumed squirrels. All predators consumed small mammals, mostly hispid cotton rats (*Sigmodon hispidus*) with highest FO for bobcats (35.0%), followed by gray fox (28.6%), and coyotes (12.4%). Beyond consumption of wild turkeys, all predators ate additional avian species with highest FO for gray foxes at 28.6%, followed by bobcats (15.0%) and coyotes (6.5%). Herpetofauna made up the majority of species found in gray fox samples with a FO of 57.1% while coyote samples only had a FO of 0.7% and bobcats consumed no herpetofauna. There were several species that we grouped into a category of “other”. We identified armadillos (*Dasypus novemcinctus*) in 13 coyote scats, cattle (*Bos taurus*) appeared in six coyote scats and invasive wild pigs (*Sus scrofa*) appeared in four coyote scats, comprising a FO of 8.5%, 3.9%, and 2.6%, respectively. Finally, we did find potential evidence of intraguild predation or scavenging; bobcats and coyotes had one sample each containing gray fox and one coyote sample contained bobcat.

**Table 2:** Number of prey species identified in predator scat using DNA metabarcoding at three sites along the Savannah River, South Carolina, USA. Scat was collected during May-June 2020–2021.

		Predator		
Diet category	Scientific name	Bobcat	Coyote	Gray fox
White-tailed deer	*Odocoileus virginianus*	5	104	0
Wild turkey	*Meleagris gallopavo*	1	14	1
Rabbit	*Sylvilagus floridanus*	7	21	0
Squirrel	Sciurus spp.	6	3	0
Small mammal	Microtus spp.	1	0	0
	*Scalopus aquaticus*	0	3	0
	*Sigmodon hispidus*	7	17	2
Avian	*Antrostomus vociferus*	0	5	0
	*Cardinalis cardinalis*	0	0	2
	*Colinus virginianus*	1	0	0
	Passiformidae	2	0	0
	*P*. *erythrophthalmus*	0	4	0
	Vireo	1	1	0
Herpetofauna	*Anolis carolinensis*	0	0	1
	*Hylidae dryophytes*	0	0	3
	*Plestiodon laticeps*	0	1	0
	*Alligator mississippiensis*	0	1	0
Other	*Bos taurus*	0	6	0
	*Dasypus novemcinctus*	0	13	0
	*Lynx rufus*	0	1	0
	*Procyon lotor*	0	1	0
	*Sus scrofa*	0	4	0
	*Urocyon cinereoargenteus*	1	1	0

#### Prey diversity and dietary overlap

All three predators behaved as specialists across sites with PDI ranging from 0.86 (bobcats) to 0.96 (coyotes). Gray foxes relied the most on a single vertebrate prey group in relation to a random selection of other diet items based on specialized diet (*d* = 0.71), followed by coyotes (0.35), and bobcats (0.34). However, coyotes had the highest species specificity index (bobcats: 0.36, coyotes: 0.52, gray foxes: 0.45), meaning that coyotes relied more heavily on a single resource than the other two predators. Bobcats exhibited the highest diversity in vertebrate diet items with Shannon diversity equaling 2.03, followed by coyotes (1.80), then gray foxes (1.52). Finally, estimates of Pianka’s niche overlap revealed low levels of vertebrate dietary overlap between gray foxes and coyotes (0.14) and gray foxes and bobcats (0.32), with moderate overlap between coyotes and bobcats (0.61).

### Coyotes among sites

#### Frequency of occurrence

**Fig 3:**
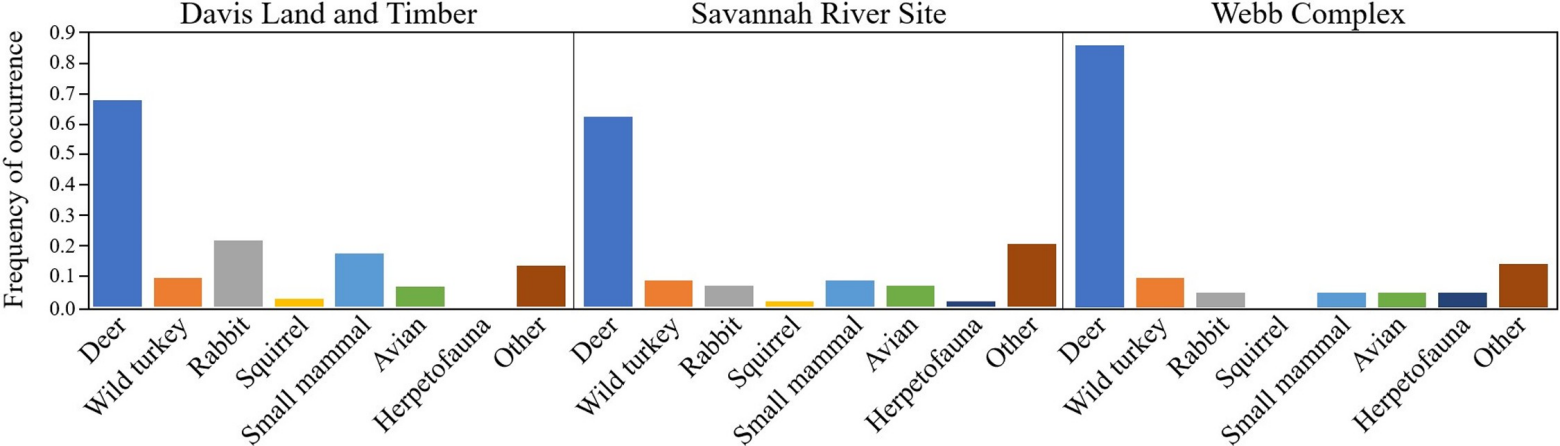
Frequency of occurrence for prey items of coyotes (*Canis latrans*) across the Davis Land and Timber, Savannah River Site, and Webb Complex study sites along the Savannah River, SC during May-June 2020–2021.

We detected no differences in vertebrate diet for coyotes among sample sites or across years except for consumption of lagomorphs among sites (χ^2^ = 7.61, *P* < 0.05). Coyote scat at the Webb Complex contained more deer with a FO of 85.7%, followed by Davis Land and Timber (67.6%), and Savannah River Site (62.1%, Fig 3). Coyotes at all three sites showed consumption of wild turkeys: scat at the Webb Complex and Davis Land and Timber contained an FO of 9.5%, followed by Savannah River Site at 8.6%. Davis Land and Timber coyote scats had greater levels of FO of lagomorphs (21.6%), followed by Savannah River Site (6.9%) and the Webb Complex (4.7%). Consumption of squirrels was only documented at the Savannah River Site (1.7%) and Davis Land and Timber (2.7%). Consumption of small mammals, including hispid cotton rats, ranged from 4.8% FO (Webb Complex) to 17.6% (Davis Land and Timber). Consumption of avian species other than wild turkey was low at all three sites (FO 4.7–6.9%). Consumption of herpetofauna only occurred in one scat sample each at Savannah River Site and the Webb Complex. All six occurrences of cattle were found at Davis Land and Timber, while invasive wild pigs were consumed at Savannah River Site and the Webb Complex. Bobcat and gray fox appeared only once each in scat collected at Davis Land and Timber.

#### Prey diversity and dietary overlap

Coyotes at all three sites during the spring behaved as specialists with paired difference indices ranging from 0.94 (Davis Land and Timber) to 0.97 (Webb Complex). Shannon diversity of vertebrate diet ranged from 1.32 at the Webb Complex to 1.74 at Davis Land and Timber. However, Davis Land and Timber exhibited the most specialization in coyotes with *d* equaling 0.13, followed by the Webb Complex (0.10) then Savannah River Site (0.09). Species-specificity indices of vertebrate diet ranged from 0.48 (Davis Land and Timber) to 0.65 (Webb Complex).

### Generalized linear mixed models

We identified eight individual clusters of scat across our three study sites: three at Davis Land and Timber, two at Savannah River Site, and three at the Webb Complex. We censored any scat outside of our 50% KDE clusters and ran GLMM models using 110 remaining samples. Use of deer and turkeys by coyotes were negatively correlated with each other (Table 3) as our top model for turkey in coyote diet included only deer FO as an explanatory variable, and conversely, our top model for deer in coyote diet included only turkey FO as an explanatory variable (Table 4). While other variables appeared in additional competitive models (ΔAICc < 2.0) for turkey and deer in coyote diet, beta estimates were not significant and tended to have high variance, potentially due to low sample size (Table 4).

**Table 3:** Parameter estimates for the top model for turkey (*Meleagris gallopavo*; top) and white-tailed deer (*Odocoileus virginianus*; bottom) consumption by coyotes (*Canis latrans*) at three sites along the Savannah River, South Carolina, U.S from May-June 2020–2021. Includes regression coefficients (β), standard error (SE), 95% confidence intervals (CI), *z*-scores, and *P*-values.

Turkey	Model variable	β	SE	95% CI	*z*	*P*
	Intercept	0.44	1.47	-2.45, 3.33	0.30	0.77
	Deer FO	-3.68	2.20	-8.00, 0.63	-1.67	0.09
Deer	Model variable	β	SE	95% CI	*z*	*P*
	Intercept	5.07	1.50	2.13, 8.01	3.38	<0.01
	Turkey FO	-32.13	10.78	-53.25, -11.01	-2.98	<0.01

**Table 4:** Top five models for turkey (*Meleagris gallopavo*; top) and white-tailed deer (*Odocoileus virginianus*; bottom) consumption by coyotes (*Canis latrans*) at three sites along the Savannah River, South Carolina, U.S from May-June 2020–2021. Includes number of variables (*k*), log-likelihood (LL), change in Akaike’s information criterion adjusted for small sample sizes (ΔAICc), and AICc weights (ω_i_). Deer = white-tailed deer frequency of occurrence (FO); Turkey = turkey FO, Rabbit = rabbit FO; Small Mammals = small mammal FO; Bird = avian FO; Forest = mean forest cover; Julian = Julian date.

Turkey	Model	*k*	LL	ΔAICc	ω_i_
	Deer	4	-36.25	0.00	0.19
	Null	3	-37.91	1.16	0.10
	Rabbit	4	-36.86	1.22	0.10
	Forest + Deer	5	-35.98	1.66	0.08
	Small Mammals	4	-37.15	1.79	0.08
Deer	Model	*k*	LL	ΔAICc	ω_i_
	Turkey	4	-54.16	0.00	0.50
	Julian + Turkey	5	-53.78	1.44	0.24
	Forest + Turkey	5	-53.99	1.87	0.20
	Global	7	-53.94	6.27	0.02
	Julian + Turkey + Rabbit + Small Mammals + Bird	8	-53.41	7.55	0.01

## Discussion

We collected scat during the springtime in South Carolina, USA to assess diet of coyotes, bobcats, and gray foxes through DNA metabarcoding. Using genetic methodologies to accurately identify the host predator of each collected sample [13], we observed a dramatic discrepancy in sample sizes between the three mesopredators, with coyote samples far exceeding either bobcat or gray fox samples. Across South Carolina, annual harvest records reveal a decline in gray fox harvest concurrent with an increase in coyote harvest since the early 1990s and consistently low bobcat harvest across years (J. Butfiloski, Furbearer and Alligator Program Coordinator, SCDNR, unpublished data). Annual scent-station furbearer surveys on the Savannah River Site from 1991–2014 also show a decline in gray fox visitations as coyote visitations increased while bobcat visitations were uniformly lower over the same timespan (M. Caudell, SCDNR, unpublished data). Additionally, although previous literature in the southeastern USA has documented limited spatial segregation between these mesopredators [2], competition with coyotes, a larger, generalist predator, may lead to competitive exclusion of bobcats and gray foxes [3, 9]. Coyote abundance, therefore, may have been higher than bobcats or gray foxes across our study sites, although we observed low levels of dietary overlap across our mesopredator guild. Finally, we sampled secondary dirt and gravel roads for scat, which may have biased our collection towards coyotes, which are known to use road systems for travel and territory marking [88]. Our comparison of diet among coyotes, bobcats, and gray foxes should be qualified by the uneven sampling success we observed among mesopredators and future research addressing dietary overlap in this predator guild should seek to maximize detection of bobcat and gray fox scat.

Contrary to our predictions, we observed low levels of dietary overlap between coyotes and gray foxes from mid-May to mid-June. The low levels of overlap were largely driven by higher levels of deer consumption by coyotes and herpetofauna consumption by gray foxes, which may have been specific to the time of year our sampling occurred and the fact that we only assessed vertebrate prey items. Because we were specifically interested in deer and turkey consumption, we focused sampling during fawning for deer [32] and nesting for turkey [67]. Previous studies have indicated that coyotes and gray foxes increase their reliance on fruit in June and July [17, 19, 20, 25, 26, 28], which coincides with the reduction of fawn availability in the region as fawn survival increases after approximately 8–10 weeks of life [32, 34]. Seasonal selection of differing prey may result in dietary partitioning among these generalist omnivores during the spring. Although we did not assess plant consumption, we predict that dietary overlap between coyotes and gray foxes may increase as coyotes begin consuming a broader range of small mammals and fruits during the rest of the year [26]. Such overlap has been documented across the sympatric range of coyotes and gray foxes and may lead to competitive exclusion in some cases [4, 9, 10, 26, 51]. Further study in the southeastern United States should examine dietary overlap between coyotes and gray foxes throughout the year, including consumption of plants, to see whether they continue to occupy different dietary niches or whether overlap increases as fawns become less susceptible to predation during the late summer [32, 34].

We documented moderate levels of dietary overlap between bobcats and coyotes across our study sites due to a shared pool of prey species. Davis Land and Timber was the one site where coyotes consumed more rabbits than at the other two sites and was privately owned containing higher percentages of managed open landcover types, which provide more suitable habitat for rabbits. Higher densities of rabbits may have increased coyote consumption of lagomorphs and could lead to increased intraguild competition with bobcats. In general, however, our findings were similar to previous diet studies, which documented higher frequencies of deer occurrence by coyotes than by bobcats and higher frequencies of rabbits, squirrels, and small mammals by bobcats than by coyotes [11, 26, 89]. Differences in diet between bobcats and coyotes likely reflect alternative hunting strategies (ambush vs. cursorial) and speak to each species occupying differing functional roles (specialist vs. generalist). Changes in relative abundance of shared prey species may increase direct competition between coyotes and bobcats as they are forced to focus to a greater extent on similar available food sources [3]. However, our study did not address coyote consumption of plant items, which would reduce dietary overlap with bobcats.

Our findings also provide insight on the impacts of mesopredator predation on game species such as white-tailed deer and wild turkey. Coyote prey-switching behavior presumably optimizes diet based on resource availability and we observed a negative relationship between FO of deer and turkeys in clusters of coyote scat. However, low sample size may have contributed to this relationship with turkey consumption by coyotes only moderately correlated with deer consumption. The fawning window of deer in this region has been documented to be up to 90 days [62] J. Kilgo, United States Forest Service, unpublished data] and newly born fawns would have been present on the landscape throughout our study period. Although increased consumption of fawns during the spring may result in lower predation on other species such as turkeys during the same time period, our data suggest that all three species are eating turkeys, specifically, and avian species, generally, more often than previously reported [12, 26, 27, 51, 52]. As we predicted, our use of DNA metabarcoding may have improved our ability to detect turkey and other avian prey in scat. However, we were unable to determine whether consumption of turkeys occurred through predation of eggs, poults, or adult turkeys. Coyotes, bobcats, and gray foxes are all known to predate adult hens, especially during the spring when nesting and brooding hens are vulnerable [35, 37, 42–44] and our sampling window may have missed predation of vulnerable hens during the first part of the reproductive period of wild turkeys in South Carolina. However, low presence of turkey in predator scat and specialization in other prey species indicate that turkeys are likely not an important component of diet in this three-predator guild.

Our data provide additional support that coyotes of the southeastern United States are specializing in deer during the spring. In fact, our finding of an FO of 68% for deer is higher than previous studies finding that coyote scat contains approximately 20–60% FO of deer [17, 19, 20, 24, 25, 52, 89]. We cannot say whether coyotes were preying on fawns, preying on adults, or scavenging. However, previous studies were able to differentiate between fawns and adult deer through comparison of guard hair sizes and reported that spring consumption of deer by coyotes was overwhelmingly focused on fawns [17, 19, 20, 24, 25, 89]. Regardless, it is clear that coyotes are preferentially consuming deer during these months. Furthermore, we did not find evidence that either Julian date or forest cover increased FO of deer in scat clusters. Instead, our data suggests that deer consumption by coyotes was ubiquitous throughout our sampling period and high across our study sites. Region-wide impacts on deer populations due to coyote predation have been thoroughly discussed over the last two decades [30, 32, 59, 90–92] with some arguing that decreases in antlerless harvest may be necessary to mitigate the effects of coyote consumption of fawns in the southeastern United States [30, 32].

Our findings document a temporal window wherein coyote vertebrate diets consist largely of white-tailed deer, resulting in a reduction of dietary overlap with other sympatric predators, most notably, gray foxes. Coyotes, bobcats, and gray foxes occupy shared space within the forested landscape of the eastern United States [1, 2]. Resource partitioning is necessary to reduce competition among coyotes, bobcats, and foxes if they are to avoid competitive exclusion, which is a growing concern for dwindling endemic populations such as the gray fox [4, 9, 10]. We recognize that our study is limited in its inference due to small sample sizes for both bobcats and gray foxes and the lack of plant and non-invertebrate diet in our analyses, but we believe that important conclusions can be drawn from our data regarding the implications of having three mesopredators sharing the southeastern United States landscape. Our findings indicate that these three species share certain similar vertebrate dietary traits during the spring (i.e., consumption of small mammal and some avian prey). However, their dietary overlap is reduced through variable consumption of specific prey items including deer for coyotes, small mammals for bobcats, and herpetofauna for gray foxes. Additionally, while coyotes prey heavily on fawns across the southeastern United States, specialization on fawns by coyotes may lessen intraguild dietary competition with the other sympatric predators.
